# Research on Optimization of RIS-Assisted Air-Ground Communication System Based on Reinforcement Learning

**DOI:** 10.3390/s25206382

**Published:** 2025-10-16

**Authors:** Yuanyuan Yao, Xinyang Liu, Sai Huang, Xinwei Yue

**Affiliations:** 1 Key Laboratory of Information and Communication Systems, Ministry of Information Industry, Beijing Information Science and Technology University, Beijing 100101, China; yyyao@bistu.edu.cn (Y.Y.); xyangliu@bistu.edu.cn (X.L.); xinwei.yue@bistu.edu.cn (X.Y.); 2 Key Laboratory of Modern Measurement & Control Technology, Ministry of Education, Beijing Information Science and Technology University, Beijing 100101, China; 3 Key Laboratory of Universal Wireless Communications, Ministry of Education, Beijing University of Posts and Telecommunications, Beijing 100876, China

**Keywords:** unmanned aerial vehicle (UAV), reconfigurable intelligent surface (RIS), deep reinforcement learning (DRL)

## Abstract

In urban emergency communication scenarios, building obstructions can reduce the performance of base station (BS) communication networks. To address such issues, this paper proposes an air-ground wireless network enabled by an unmanned aerial vehicle (UAV) and assisted by reconfigurable intelligent surfaces (RIS). This system enhances the efficacy of UAV-enabled MISO networks. Treating the UAV as an intelligent agent moving in 3D space, sensing changes in the channel environment, and adopting zero-forcing (ZF) precoding to eliminate interference from ground users. Meanwhile, joint design is performed for UAV movement, RIS phase shifts, and power allocation for users. We propose two deep reinforcement learning (DRL) algorithms, which are termed D3QN-WF and DDQN-WF, respectively. Simulation results indicate that D3QN-WF achieves a 15.9% higher sum rate and 50.1% greater throughput than the DDQN-WF baseline, while also demonstrating significantly faster convergence.

## 1. Introduction

One of the visions of sixth Generation (6G) is to realize the evolution from the Internet of Everything to the Intelligent Internet of Everything on the basis of Fifth Generation (5G), which poses great challenges to traditional terrestrial cellular networks [1]. Compared with conventional terrestrial wireless communication, Unmanned Aerial Vehicle (UAV) communication has significant advantages, such as high mobility, uninterrupted line-of-sight connection, and strong perception ability. The wireless network empowered by UAV is one of the key technologies to improve communication quality [2,3,4]. Meanwhile, UAVs can be equipped with sensing and ranging modules to accurately measure the distance between obstacles and ground equipment, ensuring flight safety, providing a sensing range, and delivering high-quality communication services [5,6].

The upcoming sixth generation (6G) driven Internet-of-Things (IoT) will face the great challenges of extremely low power demand, high transmission reliability, massive connectivities, and physical layer security [7,8,9]. To effectively address the problems of traditional communication technologies being limited by hardware costs and channel fading, Reconfigurable Intelligent Surface (RIS) has become one of the potential applications as a feasible solution to various challenges in future wireless networks. The breakthrough of RIS technology lies in transforming the communication environment into a programmable medium. Its two-dimensional metamaterial surface reconstructs electromagnetic field distribution via dynamic phase adjustment. It also dynamically regulates electromagnetic waves in a programmable manner [10]. Studies have confirmed that compared with traditional AF relays, RIS can provide substantial energy efficiency improvements for wireless networks [11,12,13]. The characteristics of RIS with low cost and UAV with high mobility have the advantages of low power consumption and high spectral efficiency. They have become important components of future communication networks. When RIS is deployed on buildings or UAV platforms, it can regulate reflection paths, change the radio environment in three-dimensional space, and achieve signal enhancement [14,15]. Moreover, studies have shown that the system network composed of RIS and UAV can obtain a more flexible network structure and a higher communication rate [16,17]. However, traditional studies have focused on the offline optimization of RIS static reflection parameters and the optimization of UAV hovering positions. With the evolution of future wireless networks, RIS-UAV communication systems need to have the ability to actively sense and reconstruct the environment.

Future communication networks will integrate various emerging technologies, which will increase the complexity of optimizing throughput and energy efficiency in the network. This complexity will bring about challenges where traditional algorithms are challenged and struggle to find optimal solutions or prove infeasible. Machine learning (ML) is widely used as a powerful tool to enhance the performance of wireless networks, especially for large-scale networks, enabling them to conduct effective optimization in dynamic environments. Due to the rapid development of ML, Deep Reinforcement Learning (DRL), as a branch in this field, provides an alternative approach to solving complex optimization problems [18,19]. DRL is a combination of neural networks and Reinforcement Learning (RL). It realizes algorithm design by using rewards provided in the environment as output values. Therefore, UAVs can be regarded as agents that acquire optimal strategies through learning from feedback, which is obtained through continuous interaction with the environment [20,21]. Therefore, the vision of future 6G and its network architecture aims to establish an intelligent communication ecosystem. By deeply integrating RIS-UAV technology with DRL, a high-efficiency air-ground integrated wireless communication network can be built to meet the needs of future intelligent communications.

### 1.1. Related Work

In recent years, research on RIS and UAV-assisted communication technologies can be mainly divided into two types: one type deploys RIS directly on UAVs as payloads to form RIS-UAV systems. The air-ground network with RIS-UAV-assisted base stations (BS) is introduced in detail in references [22,23,24,25,26]; such RIS-UAVs act as mobile airborne relays. Although the RIS-UAV scheme achieves blind area coverage, it faces significant challenges in terms of communication quality in urban emergency communications and places with high population density. The other type deploys RIS on ground buildings and uses UAVs as aerial BS. This configuration can provide ground users with better information transmission or data collection services. In comparison, through flexible deployment, the RIS-assisted BS-UAV significantly reduces the pressure on ground BS and improves the communication quality in densely populated areas. Therefore, this paper adopts the RIS-assisted BS-UAV system model to improve the performance of the system network.

Compared to traditional ground BS, UAVs are more convenient to deploy. Liu et al. utilized RIS-assisted BS-UAV and adopted a DRL approach to optimize the 2D coordinates of UAVs and reduce UAV energy consumption. This overcomes the limitation of traditional algorithms that struggle to find or cannot obtain optimal solutions in high-dimensional convex optimization [27]. Ahmad et al. adopted the prioritized experience replay method to optimize phase angles, so as to improve users’ satisfaction with the service quality of communication systems [28]. Hu et al. utilized the Double Deep Q-Network (DDQN) to optimize the 3D trajectory of UAVs, aiming to maximize the system data throughput [29]. Mei et al. proposed a method that does not require pre-prepared training data; instead, it uses environmental modeling to provide reward feedback to BS-UAVs, to optimize the UAV trajectory, and improve the communication rate [30]. Khalili et al. in [31] optimized UAV trajectory and subcarrier allocation with the help of Dueling DQN to improve the performance of heterogeneous networks supported by dual connectivity. Regarding the complex optimization problems in RIS and UAV-assisted air-ground wireless communication networks, Nguyen et al. summarized the existing DRL-based solving methods and pointed out the challenges faced by future research [32].

In the aforementioned research works, reference [25] optimize the coordinates of UAVs in a two-dimensional space, without consideration given to three-dimensional coordinate optimization. As a result, the advantages of UAVs, such as high mobility, have not been fully demonstrated. In the literature [26], although the problem of RIS phase shift optimization has been addressed, the potential of RIS has not been fully exploited by leveraging DRL. In one study [27,28], although DQN and DDQN are used to solve the problems of RIS phase shift and UAV trajectory, the optimized coordinates are still limited to two-dimensional spatial coordinates. In another work [29,30,31], although the DRL method is adopted, power discretization or the failure to consider power allocation will lead to an overall decline in network performance. However, traditional DRL algorithms with discrete action spaces have significant limitations: the value overestimation phenomenon in DQN and Dueling DQN can cause decision biases in agents when channel states change abruptly, while the Q-value oscillation problem in DDQN restricts the stability of UAV trajectory planning. To address these bottlenecks in agent actions, the Dueling Double Deep Q-Network (D3QN) has achieved breakthroughs through innovative architectural integration. Its core idea is to combine the decomposed structure of state value and advantage value from Dueling DQN with the dual-network structure of DDQN. This enables it to accurately estimate the Q-values of all actions of the agent, effectively mitigating the overestimation problem, and boasts the advantages of fast convergence speed and stable convergence effect.

Therefore, the deep reinforcement learning framework is integrated into the RIS-assisted UAV communication model to construct collaborative optimization under a multi-dimensional action space. The sum-rate expression for multiple users under this model is established, and an optimization variable algorithm based on the D3QN framework (Dueling Double Deep Q-Network water-filling algorithm, D3QN-WF) is proposed. RIS changes its phase to reconstruct the channel state; UAVs perceive environmental changes and adjust their 3D coordinates through decision-making; and by optimizing the transmission power of UAVs, the system sum-rate is maximized, and the throughput during communication is improved.

### 1.2. Contributions

- A joint optimization framework for RIS-assisted UAV air-to-ground wireless communication networks under 3D spatial coordinates is proposed. This framework significantly improves the network system sum-rate and throughput during communication by collaboratively optimizing the 3D spatial coordinates of UAVs, the RIS phase shift matrix, and the UAV transmission power, thus giving full play to the synergistic potential of RIS and UAVs;
- A joint optimization method combining BS transmission power optimization based on the water-filling algorithm and D3QN. Aiming at the convexity problem of BS transmission power optimization, the water-filling algorithm is adopted for an efficient solution; meanwhile, the D3QN algorithm is innovatively used to solve the optimization problem in the discrete action space. It jointly optimizes the 3D coordinates of UAVs and RIS phases, effectively overcoming the limitations of traditional methods in high-dimensional non-convex optimization problems;
- Detailed verification results are provided to demonstrate the effectiveness of the proposed algorithm in improving the system sum-rate and throughput. Simulation results show that the proposed algorithm has significant advantages in rate improvement. In addition, compared with the DDQN-WF, the D3QN-WF algorithm shows obvious advantages in handling multi-dimensional action spaces, with faster convergence and higher stability. This method increases the system sum-rate by 15.9% and the throughput by 50.1%, providing new ideas for the dynamic optimization of future intelligent communication networks.

## 2. System Model

**Figure 1:**
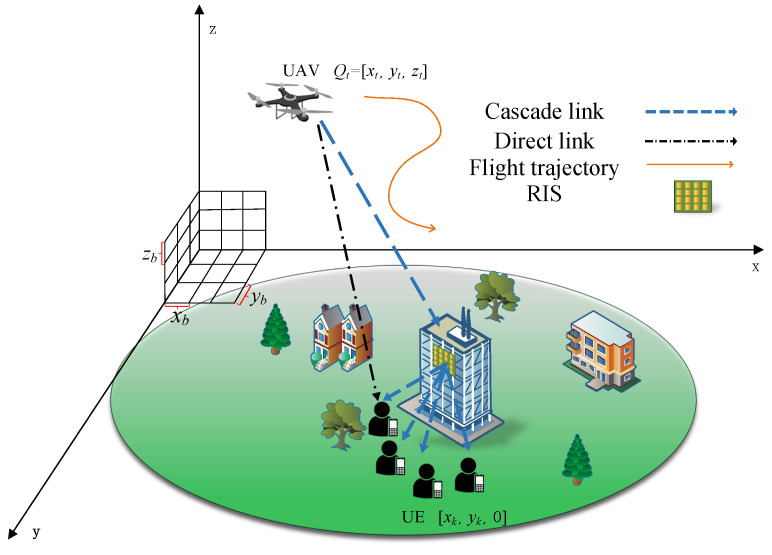
System model for RIS-Assisted UAV-BS air-to-ground downlink communications network.

Figure 1 depicts a RIS-assisted BS-UAV air-to-ground intelligent communication network system. An RIS with *N* reflecting elements is deployed on a high-rise building to facilitate the reconstruction of the channel environment. The UAV equipped with *M* antennas is regarded as BS, which communicates with single-antenna users, and it has a sensing antenna at its bottom to sense ground users, with a maximum sensing elevation angle of ω. By perceiving the environment, associating with users, and collecting real-time channel state information (CSI), the UAV makes adjustments to its 3D position. When a UAV detects users, there are direct links and cascaded links between the UAV and the users. The direct link includes both line-of-sight (LoS) and non-line-of-sight (NLoS) links. For the cascaded link, only LoS link exists between the UAV and the RIS, while both LoS and NLoS links exist between the RIS and the users. When the users are outside the detection range of the UAV, the RIS is used to cover the blind area. In this case, only the cascaded link exists between the UAV and the ground users.

It is assumed that the system’s Area of Interest (AoI) is discretized into multiple cells of equal size, and the coordinates of the center of cell *i* can be expressed as $L_{i}^{c}=\left[x_{i},y_{i},z_{i}\right]\in R^{3\times 1}$. The variables $x_{b}$,$y_{b}$, and $z_{b}$ represent the distances between adjacent cells along the *x*-axis, *y*-axis, and *z*-axis, respectively. The total time slots of the system can be expressed as *T*, and the three-dimensional coordinates of the UAV at time slot *t* can be expressed as $Q_{t}=\left[x_{t},y_{t},z_{t}\right]\in L^{c}$, where t∈{1,2,…,T}. Ground users move within a small range and are evenly distributed on one side of the RIS. Their coordinates can be expressed as ($x_{k}^{t}$, $y_{k}^{t}$, $z_{k}^{t}$). In communication networks under multi-target scenarios, the constraint on the UAV’s sensing range is a key factor to be considered in practice. In each time slot, the UAV needs to select targets within its coverage area from all potential IoT devices according to its current flight path for data transmission operations. A sensing communication variable $a_{k}$ is defined, and its sensing coverage is related to the height and angle of the UAV.

(1) $$a_{k}=\left\{\begin{array}{cc} 1 & \;R\leq H\tan\omega \\ 0 & \;R>H\tan\omega \end{array}\right.$$

where *R* is the horizontal distance between the UAV and the user, ω represents the sensing angle and *H* represents the height of the UAV. When $a_{k}=1$, the user is within the sensing range.

### 2.1. Channel Model

The UAV acting as a BS moves gradually toward the users. When the user is within the UAV’s sensing range, the channel gain of the direct link between the aerial BS and the ground user *k* can be expressed as $h_{u,k}^{H}\in C^{1\times M}$ which can be modeled as a Rician channel, expressed as:

(2) $$h_{u,k}^{H}=\sqrt{\beta d_{u,k}^{-\alpha }}\left(\sqrt{\frac{\hat{R}}{1+\hat{R}}}h_{u,k}^{LoS^{H}}+\sqrt{\frac{1}{1+\hat{R}}}h_{u,k}^{NLoS^{H}}\right)$$

where $h_{u,k}^{LoS^{H}}$ and $h_{u,k}^{NLoS^{H}}$ are used to represent the fast fading component for LoS path and NLoS path between the UAV and the user *k*, respectively. $\hat{R}$ is the Rician factor. The path loss between the BS and *k*-th user is given by $\sqrt{\beta d_{u,k}^{-\alpha }}$, where β denotes the channel gain at a 1 m reference distance, α≥2 is the path loss exponent, and $d_{u,k}$ is the distance between the UAV and user *k*. For the cascaded channel, there are two links: the UAV-RIS link and the RIS-User link. In the UAV-RIS link, only the LoS link $H_{u,r}^{H}\in C^{N\times M}$ exists, which can be expressed as:

(3) $$H_{u,r}^{H}=\sqrt{\beta d_{u,r}^{-\alpha }}\sqrt{\frac{\hat{R}}{1+\hat{R}}}H_{u,r}^{LoS^{H}}$$

$H_{u,r}^{LoS^{H}}\in C^{N\times M}$ is the fast fading component of the LoS channel between UAV and RIS; $d_{u,r}$ is the Euclidean distance. Secondly, both LoS and NLoS propagations exist in the RIS-User link. Therefore, it is also modeled using a Rician channel, denoted $h_{r,k}^{H}\in R^{1\times N}$. The channel gain of the RIS-User link is as follows:

(4) $$h_{r,k}^{H}=\sqrt{\beta d_{r,k}^{-\alpha }}\left(\sqrt{\frac{\hat{R}}{1+\hat{R}}}h_{r,k}^{LoS^{H}}+\sqrt{\frac{1}{1+\hat{R}}}h_{r,k}^{NLoS^{H}}\right)\;$$

### 2.2. Downlink Signal Transmission Modeling and Optimization

Consider that the UAV transmits linear preambles, and the transmitted signal is:

(5) $$x=\sum_{k=1}^{K}\sqrt{p_{k}}w_{k}s_{k}$$

where $p_{k}$, k∈K is the transmit power allocated by the UAV to user *k*, $s_{k}$ is the unit-power complex transmit symbol, and $w_{k}\in C^{M\times 1}$ is the precoding direction vector of the *k*-th user. When the UAV can detect the user, the communication quality is improved through the direct link. When the user is not within the UAV’s sensing range, the blind area is compensated through the cascaded link of the RIS. Therefore, the signal received by the *k*-th user can be expressed as:

(6) $$y_{k}=\left(a_{k}h_{u,k}^{H}+h_{r,k}^{H}\Phi H_{u,r}^{H}\right)\sum_{i=1}^{K}\sqrt{p_{i}}w_{i}s_{i}+\sigma^{2}$$

where the RIS phase shift matrix is denoted by $\Phi ≜diag[\phi_{1},\phi_{2},\ldots ,\phi_{N}]$. For each element in the diagonal matrix Φ, that is, the phase angle of the *n*-th reflecting element is denoted as $\phi_{n}=e^{j\theta_{n}},\forall n=1,2,\ldots ,N$,$\theta_{n}\in \left[0,2\pi \right]$. Where $\sigma^{2}∼CN\left(0,\sigma^{2}\right)$ is the additive white Gaussian noise. According to Formula (6), the received signal interference noise ratio (SINR) at the *k*-th user in the system can be expressed as:

(7) $${\hat{\gamma }}_{k}≜\frac{p_{k}{\left|\left(a_{k}h_{u,k}^{H}+h_{r,k}^{H}\Phi H_{u,r}^{H}\right)w_{k}\right|}^{2}}{\sum_{i=1,i\neq k}^{K}p_{i}{\left|\left(a_{i}h_{u,i}^{H}+h_{r,i}^{H}\Phi H_{u,r}^{H}\right)w_{i}\right|}^{2}+\sigma^{2}}$$

where $\sum_{i=1,i\neq k}^{K}p_{i}{\left|\left(a_{i}h_{u,i}^{H}+h_{r,i}^{H}\Phi H_{u,r}^{H}\right)w_{i}\right|}^{2}$ is the interference signal power received by user *k*. The UAV departs from the starting point, plans its trajectory, and searches for the optimal deployment position that maximizes the system communication performance. For the sake of fairness, each user is allocated a bandwidth of *B*. Therefore, the objective function for optimizing the system sum rate under the UAV’s deployment position can be expressed as:

(8)P1:maxP,Φ,QR≜∑k=1KBlog21+γ^k(8a)s.t.ak∈0,1(8b)ejθn=1,∀n=1,2,…,N(8c)∑k=1Kpk=P(8d)pk≥0,∀k=1,2,…,K(8e)xmin≤xt≤xmax,∀t=1,2,…,T(8f)ymin≤yt≤ymax,∀t=1,2,…,T(8g)zmin≤zt≤zmax,∀t=1,2,…,T(8h)∥xt+1−xt∥2≤xb,∀t=1,2,…,T−1(8i)∥yt+1−yt∥2≤yb,∀t=1,2,…,T−1(8j)∥zt+1−zt∥2≤zb,∀t=1,2,…,T−1

where the constraint (8a) represents the sensing state of the direct link between the UAV and the *k*-th user; (8b) ensures that the RIS reflection unit only changes the phase of the signal without altering its amplitude; (8c) ensures that the transmit power of the BS after encoding is equal to *P*; (8d) ensures that when optimizing power, the user’s power is not lower than the minimum value of 0; (8e) to (8g) limit the 3D coordinates of the UAV’s flight to prevent it from flying out of the boundary; (8h) to (8j) ensure the displacement limit of the UAV in each time slot. Since the optimization of the UAV trajectory coordinates Q and the RIS phase matrix θ is covered in Section 3, this subsection only discusses in detail the user’s power P in Formula (8); in order to suppress channel interference in multi-user scenarios, the zero-forcing (ZF) coding algorithm in linear coding is adopted to eliminate signal interference between users [27]:

(9) $$\left(a_{i}h_{u,i}^{H}+h_{r,i}^{H}\Phi H_{u,r}^{H}\right)w_{k}=0,\;\forall i\neq k,\;i\in K.$$

$h_{k}^{H}=a_{k}h_{u,k}^{H}+h_{r,k}^{H}\Phi H_{u,r}^{H}$ is used to represent the direct and cascaded channel gains of user *k*, where $h_{k}^{H}\in C^{1\times M}$. Under this condition, the global channel matrix can be written as $H^{H}={\left(h_{1},\ldots ,h_{k}\right)}^{H}$, where $H\in C^{M\times K}$. Therefore, the transmit precoding matrix can be expressed as $W=\left(w_{1},\ldots ,w_{k}\right)$, where $W\in C^{M\times K}$. Under the condition of (9), the zero-force(ZF) precoding matrix can be solved through $H^{H}W=E$, and W can be expressed as:

(10) $$W=H{\left(H^{H}H\right)}^{-1}$$

W is the right pseudoinverse of $H^{H}$. After ZF precoding, there is no interference between users. To solve the optimal power allocation, the beam vector $w_{k}$ of the *k*-th user is normalized in the transmit precoding matrix W.

(11) $$w_{k}^{*}=\frac{w_{k}}{{∥w_{k}∥}^{2}}$$

according to Formula (11), $w_{k}^{*}$ is the beam direction of user *k*, and $p_{k}$ is the transmit power allocated to user *k*, therefore $\sum_{k=1}^{K}{∥\sqrt{p_{k}}w_{k}^{*}∥}^{2}=P$. Then, the SINR of the *k*-th user is $\gamma_{k}=\frac{p_{k}{\left|\left(a_{k}h_{u,k}^{H}+h_{r,k}^{H}\Phi H_{u,r}^{H}\right)w_{k}^{*}\right|}^{2}}{\sigma^{2}}$ Since the bandwidth allocated among users is the same, the power allocation part can further equate the optimization problem to:

(12)P2:−∑k=1Kln1+pkξkσ2(12a)s.t.∑k=1Kpk=P(12b)pk≥0,∀k=1,2,…,K

where $\xi_{k}={\left|\left(a_{k}h_{u,k}^{H}+h_{r,k}^{H}\Phi H_{u,r}^{H}\right)w_{k}^{*}\right|}^{2}$, this problem can be solved using the water-filling algorithm to allocate the transmit power to each user, and its Lagrangian function is:

(13) $$L\left(p_{k},v_{k},\mu \right)=-\sum_{k=1}^{K}\ln\left(1+\frac{P_{k}\xi_{k}}{\sigma^{2}}\right)-\sum_{k}^{K}v_{k}p_{k}+\mu \left(\sum_{k=1}^{K}p_{k}-P\right)$$

$v_{k}$ is the Lagrange multiplier corresponding to the inequality constraint $p_{k}\geq 0$, and μ is the Lagrange multiplier corresponding to the equality $\sum_{k=1}^{K}p_{k}=P$. The KKT necessary conditions for this optimization problem can be expressed as:

(13a)pk*≥0(13b)∑k=1Kpk*=P(13c)vk*≥0(13d)vk*pk*=0(13e)∂Lpk,vk,μ∂pkpk*,vk*,μ*=−ξkσ2+ξkpk*−vk*+μ*=0

where $p_{k}^{*}$ is the optimal power allocation, $v_{k}^{*}$ and $\mu^{*}$ are the optimal Lagrange multipliers. According to (13c) and (13e), the following inequalities can be obtained:

(14) $$\mu^{*}\geq \frac{\xi_{k}}{\sigma^{2}+\xi_{k}p_{k}^{*}},k=1,\ldots ,K$$

When $\mu^{*}<\frac{\xi_{k}}{\sigma^{2}}$, it can be derived by combining (14) that

(15) $$\frac{\xi_{k}}{\sigma^{2}+\xi_{k}p_{k}^{*}}\leq \mu^{*}<\frac{\xi_{k}}{\sigma^{2}}$$

It can be solved from (13a) and (15) that:

(16) $$p_{k}^{*}\geq \frac{1}{\mu^{*}}-\frac{\sigma^{2}}{\xi_{k}}$$

According to the complementary slackness condition in (13d), if $p_{k}^{*}=0$, the derived formula from (14) would contradict $\mu^{*}<\frac{\xi_{k}}{\sigma^{2}}$, Therefore, when $p_{k}^{*}>0$, it can be obtained from (13e) that $v_{k}^{*}=0$, so the following equality holds:

(17) $$\mu^{*}-\frac{\xi_{k}}{\sigma^{2}+\xi_{k}p_{k}^{*}}=0$$

Therefore, when $\mu^{*}<\frac{\xi_{k}}{\sigma^{2}}$, there is $p_{k}^{*}>0$, and the closed-form solution can be obtained from (16) and (17):

(18) $$p_{k}^{*}=\frac{1}{\mu^{*}}-\frac{\sigma^{2}}{\xi_{k}},\;\mu^{*}<\frac{\xi_{k}}{\sigma^{2}}$$

When $\mu^{*}\geq \frac{\xi_{k}}{\sigma^{2}}$, if $p_{k}^{*}>0$, according to the complementary slackness condition in (13d), there is $v_{k}^{*}=0$. From (17) and $\mu^{*}\geq \frac{\xi_{k}}{\sigma^{2}}$, it can be solved that $p_{k}^{*}\leq 0$, which contradicts $p_{k}^{*}>0$. According to (13a), when $\mu^{*}\geq \frac{\xi_{k}}{\sigma^{2}}$ the only feasible solution is $p_{k}^{*}=0$. Therefore, the optimal power allocation can be expressed as:

(19) $$p_{k}^{*}=\left\{\begin{array}{cccc} \; & \frac{1}{\mu^{*}}-\frac{\sigma^{2}}{\xi_{k}},\; & \; & \mathrm{if}\;\mu^{*}<\frac{\xi_{k}}{\sigma^{2}}\\ \; & 0,\; & \; & \mathrm{if}\;\mu^{*}\geq \frac{\xi_{k}}{\sigma^{2}} \end{array}\right.$$

**Figure 2:**
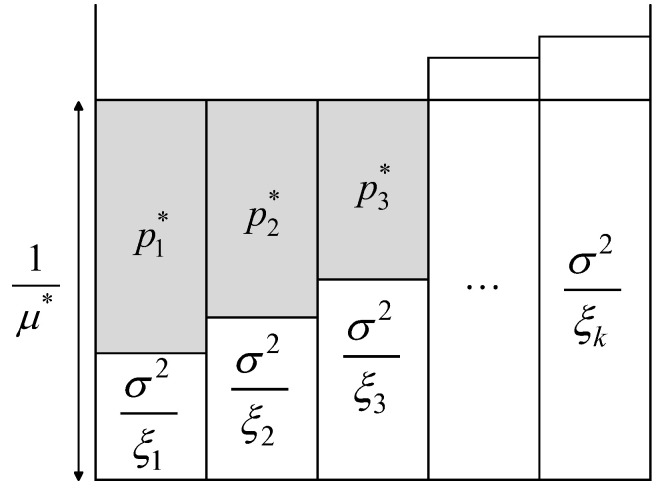
Classic water filling power distribution method.

The above process can be described as pouring the total power *P* into a water tank, and the classic method shown in Figure 2 can be used for power allocation.

Where $\frac{1}{\mu^{*}\left(i\right)}$ represents the water level. The channel gains of the *K* users are arranged in descending order. The water level can be derived from (13a) and the channel state information as follows:

(20) $$\frac{1}{\mu^{*}\left(i\right)}=\frac{P+\sum_{k=1}^{K-i+1}\frac{\sigma^{2}}{\xi_{k}}}{K-i+1}.$$

where i=1…K; k=1…K−i+1. After obtaining the water level from (20), the power allocation for each user can be calculated using (19). By adjusting the UAV transmit power $P=(p_{1},\ldots ,p_{k})$, the RIS unit phase shift matrix Φ, and the UAV trajectory Q, the maximum system sum rate can be achieved. First, the UAV perceives channel changes and uses ZF coding to eliminate inter-user interference, then employs the water-filling algorithm to solve for the optimal power allocation P among users. Then the RIS unit phase shift θ and UAV trajectory *Q* are included in Equation (8). This problem is non-convex; therefore, a DRL-based D3QN-WF algorithm is designed in the third subsection to solve it.

### 2.3. Throughput Model

To fully demonstrate the advantages and potential of the D3QN-WF algorithm, this study will calculate the total throughput of the UAV during the entire communication period based on the system sum rate analyzed in Formula (8). The throughput over all *T* time slots is defined as follows [24]:

(21) $$R_{sum}=\sum_{t=1}^{T}R\left(t\right)$$

where *R*(*t*) is the system sum rate under the UAV’s deployment position in the *t* time slot.

## 3. DRL-Based Algorithms

Jointly adjusting the 3D coordinates Q of the UAV and the phase angles θ of the RIS to maximize the system sum rate can be categorized as a Markov Decision Process (MDP), which consists of states, actions, rewards, and state transition probabilities:

- State $s_{t}$: The state of the system at time slot *t* is defined as $s_{t}=\left(Q_{t},\Phi_{t}\right)$, $s_{t}\in S$, *S* is denoted as the state space. where $Q_{t}$ represents the 3D coordinates of the UAV at time slot *t*. $\Phi_{t}$ denotes the phase shift matrix of the RIS at time slot *t*.
- Action $a_{t}$: Define the spatial action of the UAV in the system at time slot *t*: The spatial action of the UAV includes horizontal movement and vertical movement. The phase matrix of the RIS elements is dynamically optimized, with the phase parameter of each RIS element being discretely adjusted within a predetermined discretization range. The action $a_{t}$ belongs to the action space *A*.
- State Transition Probability $p\left(s_{t+1}\left|s_{t},a_{t}\right.\right)$: The optimization problem of UAV path planning and RIS phase angle adjustment can be simplified as an MDP. The state transition probability depends only on the current state $s_{t}$ and action $a_{t}$.
- Reward $r_{t}$: The UAV receives feedback on its actions from the environment. Rewards can help it evaluate the quality of its actions and adjust its strategy accordingly. This strategy is adjusted according to the magnitude of the reward to obtain higher returns, thereby improving the sum rate of the system. $r_{t}$ denotes the reward obtained by the system after taking action $a_{t}$ under state $s_{t}$ at time slot *t*. Thus, the reward $r_{t}$ is the downlink sum rate R(t) of the system at time slot *t*, as shown follows:
  (22) $$r_{t}=R\left(t\right)$$
  The goal of MDP is to maximize the cumulative expected return. When the system is in the optimal state, when the maximum rate is achieved, the maximum return is obtained.

To provide a detailed introduction to D3QN, we first present the basic structure of DRL algorithms. In reinforcement learning, Q-learning is an effective method for solving an MDP. It constructs an action-value function $Q(s_{t},a_{t})$ to evaluate the quality of taking action $a_{t}$ in state $s_{t}$. For a state: under a given policy, $Q(s_{t},a_{t})$ is defined as the sum of future rewards:

(23) $$Q\left(s_{t},a_{t}\right)=r_{t}+\gamma \cdot r_{t+1}+\ldots +\gamma^{T-t-1}\cdot r_{T-1},\;0\leq t\leq T-1\;,$$

where γ∈[0,1] is a discount factor that measures the importance of current and future rewards. $Q(s_{t},a_{t})$ represents the expected cumulative reward obtained by taking action $a_{t}$; in state $a_{t}$, the optimal action-value function $Q^{*}\left(s_{t},a_{t}\right)$ is defined as:

(24) $$Q^{*}\left(s_{t},a_{t}\right)=\max Q\left(s_{t},a_{t}\right)$$

and it satisfies the Bellman optimality equation:

(25) $$Q^{*}\left(s_{t},a_{t}\right)=r_{t}+\gamma \cdot \max_{a_{t+1}\in A}\;Q^{*}\left(s_{t+1},a_{t+1}\right)$$

by selecting the action with the highest value in state $s_{t}$, the optimal policy can be derived from the optimal action-value function. The update rule for $Q(s_{t},a_{t})$ is as follows:

(26) $$Q\left(s_{t},a_{t}\right)=Q\left(s_{t},a_{t}\right)+\bar{\alpha }\cdot \left[r_{t}+\max_{a_{t+1}\in A}\;Q\left(s_{t+1},a_{t+1}\right)-Q\left(s_{t},a_{t}\right)\right]\;$$

where $\bar{\alpha }$ denotes the learning rate. Θ and $\Theta^{-}$ represent the weights of the estimation network and the target network, respectively. The estimation network is used to obtain $Q\left(s_{t},a_{t}\left|\Theta \right.\right)$, which approximates $Q(s_{t},a_{t})$. During the training process, the weights Θ of the estimation network only need to be updated by minimizing the loss function L(Θ):

(27) $$L\left(\Theta \right)={\left({y_{t}}^{DQN}-Q\left(s_{t},a_{t}\left|\Theta \right.\right)\right)}^{2}$$

(28) $${y_{t}}^{DQN}=r_{t}+\max_{a_{t+1}\in A}\;Q\left(s_{t+1},a_{t+1}{\left|\Theta \right.}^{-}\right)$$

where ${y_{t}}^{DQN}$ is the target value. The weights $\Theta^{-}$ of the target network are updated periodically with the weights Θ of the estimation network, with an update interval of *O* steps. Since DQN directly selects target actions based on target Q-values, it has the problem of overestimation. To solve this problem, DDQN decouples the two steps of selecting target actions and calculating target Q-values through the loss function L(θ):

(29) $$L\left(\Theta \right)={\left({y_{t}}^{DDQN}-Q\left(s_{t},a_{t}\left|\Theta \right.\right)\right)}^{2}$$

(30) $$L\left(\Theta \right)={y_{t}}^{DDQN}=r_{t}+Q\left(s_{t+1},\underset{a_{t+1}\in A}{\arg\max}\;Q\left(s_{t+1},a_{t+1}\left|\Theta \right.\right){\left|\Theta \right.}^{-}\right)\;$$

when the state is at $s_{t}$, the operation of estimating the action value function $Q(s_{t},a_{t})$ using DQN or DDQN will lead to unstable output of the value function. To solve the MDP problem, D3QN is used to improve training results. The architecture of Dueling DQN utilizes a sequence of two fully connected layers, which allows the state value and the optimal action to be estimated, respectively. This design allows the neural network to make a basic judgment on a given state, and then revise its judgment through different actions $a_{t}$. On the other hand, Dueling DQN outputs a *Q* function, which can be combined with DDQN to eliminate the widespread overestimation problem in traditional DQN algorithms.

**Figure 3:**
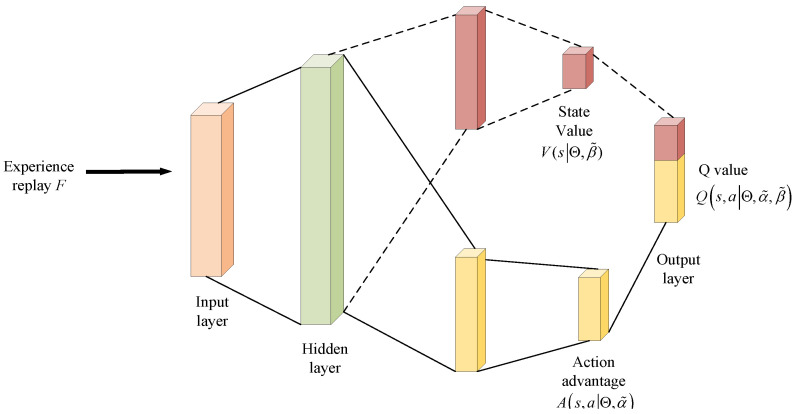
The D3QN architecture.

**Figure 4:**
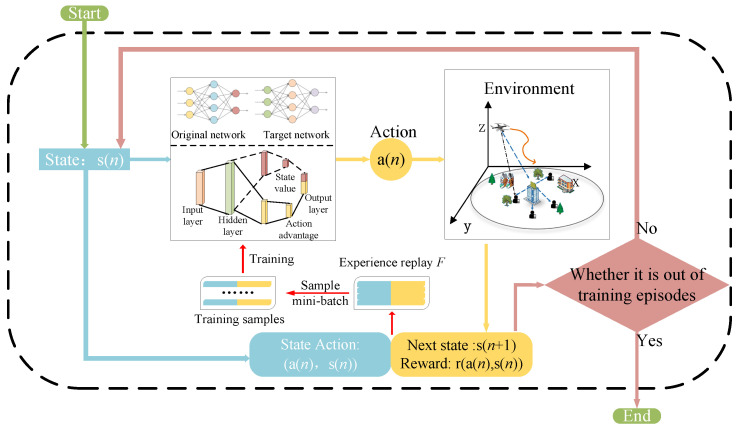
Flowchart of D3QN algorithm.

The network structure of D3QN is shown in Figure 3. First, empirical parameters of size F are placed in the input layer. After passing through the hidden layer, effective features are extracted and sent to two separate paths, respectively. One path is the state value, which is irrelevant to the actions to be taken by the UAV and the RIS. The output of this part represents the value reflecting the current environment. This function is used to evaluate the value of UAV and RIS in a specific environment, and is called the value function V(s). The other path focuses on action value, expressed as the advantage function A(s,a); it is used to measure the relative advantage of each action selected by UAV and RIS from *A*. These two paths are denoted as $V(s\left|\Theta ,\right.\tilde{\beta })$ and $A(s,a\left|\Theta ,\tilde{a}\right.)$, where θ, $\tilde{\alpha }$ and $\tilde{\beta }$ represent the parameters of the hidden layer (DNNs), the value function network, and the advantage function network, respectively. D3QN independently learns the value and the advantage of actions, then combines them to form the output layer, which outputs $Q(s_{t},a_{t})$. This can be further expressed as $Q\left(s,a\left|\Theta ,\tilde{\alpha },\tilde{\beta }\right.\right)$. Combining the action advantage A(s,a) and the state value V(s), while subtracting the average value of the action advantage A(s,a), the equation is as follows:

(31) $$Q\left(s_{t},a_{t}\left|\Theta ,\tilde{\alpha },\tilde{\beta }\right.\right)=V\left(s_{t}\left|\Theta ,\right.\tilde{\beta }\right)+\left(A\left(s_{t},a_{t}\left|\Theta ,\tilde{\alpha }\right.\right)-\frac{1}{\left|A\right|}\sum_{a_{t+1\in A}}A\left(s_{t},a_{t+1}\left|\Theta ,\tilde{\alpha }\right.\right)\right)\;$$

where $\left|A\right|$ represents the dimension of the action space *A*. Additionally, the loss function of D3QN can be expressed as:

(32) $$L\left(\Theta ,\tilde{\alpha },\tilde{\beta }\right)={\left({y_{t}}^{D3QN}-Q\left(s_{t},a_{t}\left|\Theta \right.,\tilde{\alpha },\tilde{\beta }\right)\right)}^{2}$$

(33) $${y_{t}}^{D3QN}=r_{t}+\gamma Q\left(s_{t+1},\underset{a_{t+1}\in A}{\arg\max}\;Q\left(s_{t+1},a_{t+1}\left|\Theta ,\tilde{\alpha },\tilde{\beta }\right.\right)\left|\Theta^{-},{\tilde{\alpha }}^{-},{\tilde{\beta }}^{-}\right.\right)\;$$

where $\Theta^{-}$, ${\tilde{\alpha }}^{-}$, and ${\tilde{\beta }}^{-}$ represent the parameters of the target network, which are periodically copied and updated according to θ, $\tilde{\alpha }$, and $\tilde{\beta }$ in the estimation network. The process of D3QN is shown in Figure 4.

The process of the D3QN algorithm is illustrated in Figure 4. Each episode consists of two stages: the exploration stage (Steps 3 to 24 in Algorithm 1) and the training stage (Steps 25 to 27). In the exploration stage, Action a(n) is selected via a random approach (with probability ε) or the greedy approach in Step 5 to obtain the UAV trajectory and RIS phase shifts. Meanwhile, the UAV is restricted from flying out of the defined range, and the next state s(n+1) is acquired. Then, ZF precoding and the WF algorithm are used for power allocation (Steps 8 to 20), and the corresponding reward r(a(n),s(n)) is calculated. Subsequently, the newly generated sample (s(n),a(n),r(n),s(n+1)) is stored in the experience replay buffer F in Step 23. In the training stage, a random mini-batch of samples is selected from the experience replay buffer to train the online network Q(·) and the target network $Q^{-}\left(\cdot \right)$. After that, the target value $y_{t}^{D3QN}$ from Equation (33) is used to update the weights Θ of the online network by minimizing the loss $L\left(\Theta \right)$, which follows the general definition in Equation (32).

The state space: The system state $s_{t}\in S$ at time slot *t* includes the 3D coordinates $\left(x_{t},y_{t},z_{t}\right)$ of the UAV, the phase $\theta_{nt}$ of the *n*-th reflecting element in the RIS. Thus, $S_{t}$ can be defined as: $S_{t}=\left(x_{t},y_{t},z_{t},\theta_{nt}\right)$

Action: Based on the observed environmental state $S_{t}$, the UAV selects an action from the action space *A* to execute. Among them, A consists of three parts: (1) the horizontal movement of the UAV $\Delta L_{UAV}\in \left\{\left(x_{b},0\right),\left(-x_{b},0\right),\left(0,y_{b}\right),\left(0,-y_{b}\right),\left(0,0\right)\right\}$; (2) The vertical movement of the UAV: $\Delta H_{UAV}\in \left\{\left(z_{b},-z_{b},0\right)\right\}$. Since the water-filling algorithm can obtain the optimal UAV transmission power, the action space *A* does not include the BS transmission power. Instead, the BS transmission power is placed in the environment, and after being solved, it is treated as the observed value of the environment. (3) The phase shift of each reflective element in the RIS $\Delta \theta_{n}\in \left\{\frac{\pi }{80},-\frac{\pi }{80},0\right\}$.
**Algorithm 1:** D3QN-WF algorithm
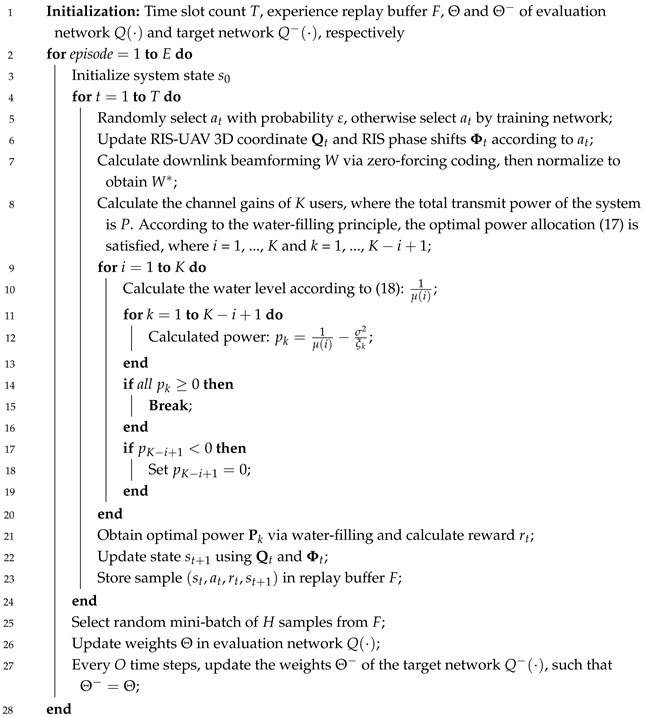


Reward: By taking actions, the 3D coordinates of the UAV and the phase shift matrix of the RIS are changed. Then, the objective function (12) is solved in the environment, the optimal power allocation (19) is obtained using the water-filling algorithm, and the optimal system sum rate of time slot *t* is calculated. The reward of time slot *t* is the same as that in Equation (22). The pseudocode of the algorithm D3QN-WF is shown in Algorithm 1.

## 4. Complexity of Simulation Analysis

The complexity of the D3QN-WF algorithm can be attributed to two aspects: environmental complexity and training process complexity. In this environment, the water-filling algorithm handles the allocation of BS transmission power to *K* users, with the number of optimization variables being *K*. The complexity of the water-filling algorithm is denoted as $f_{\mathrm{wf}}$. Then, the computational complexity for the BS to calculate the transmission power in each iteration is $f_{\mathrm{wf}}K$. During the training process, the complexity of the D3QN-WF algorithm is $O\left(\left|S\right|\cdot \left|A\right|\right)$. Where $\left|S\right|$ and $\left|A\right|$ denote the sizes of the state space and action space, respectively. Therefore, the computational complexity of D3QN-WF is: $O\left(\left|S\right|\cdot \left|A\right|\cdot f_{\mathrm{wf}}K\right)$.

## 5. Simulation Result

In this section, the D3QN-WF algorithm is applied to an RIS-assisted BS-UAV wireless communication network. Comparisons are made with methods such as D3QN with average power, D3QN with unoptimized phase angles, DDQN-WF, and the D3QN-Majorize-Minimization algorithm(D3QN-MM) [11] that optimizes phase using convex optimization methods. Furthermore, the impact of RIS with different numbers of reflecting elements on the system rate is analyzed within the D3QN-WF algorithm. The system simulation parameters are presented in Table 1. The hyperparameters in DRL are shown in Table 2. Python 3.7.12 is used to build a DNN based on TensorFlow 2.0.0 for the simulations.

Assume the UAV starts at (—300 m, —300 m, 150 m), UEs are randomly distributed on the ground within (0 m~100 m, 0 m~200 m, 0 m), and the RIS is located at (0 m, 150 m, 20 m). The UAV’s area of interest (AoI) spans (—300 m~300 m, —300 m~300 m, 50 m~150 m), and the grid step sizes are $x_{b}$ = $y_{b}$ = $z_{b}$ = 5 m.

**Figure 5:**
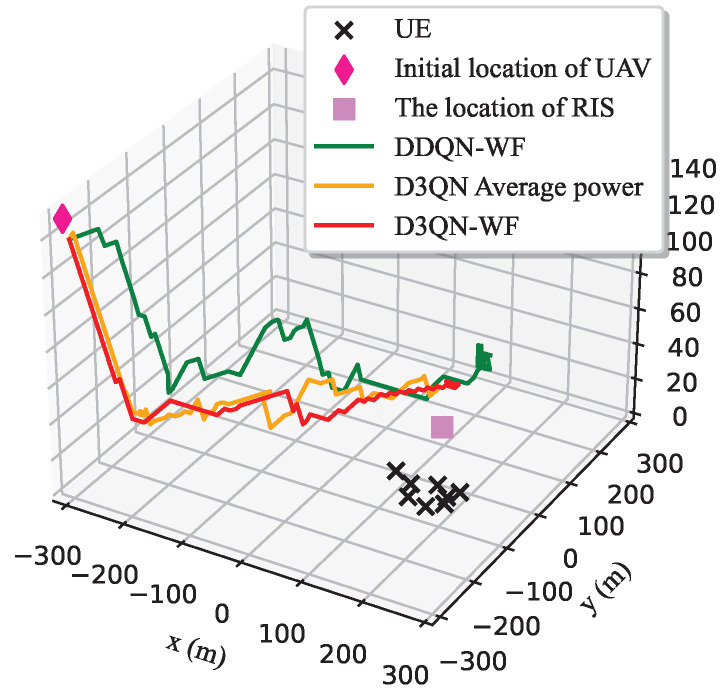
3D spatial coordinates.

**Figure 6:**
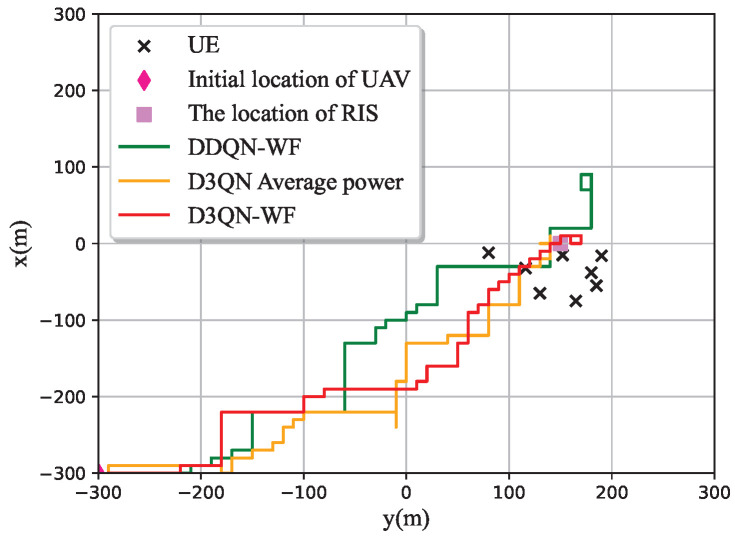
2D spatial coordinates.

Figure 5 and Figure 6 compare the 3D flight trajectories of the UAV and their corresponding 2D projections for three algorithms: DDQN-WF, D3QN-WF, and D3QN with average power. Starting from the initial position (—300 m, —300 m, 150 m), the UAV perceives communication users gradually, searches for the optimal trajectory, and globally optimal deployment position, then hovers near this position until the end of the time slot. This hovering and lingering behavior occurs because no termination condition is set in the DRL algorithm, leading to unrestricted movement throughout the entire time slot period *T*. If the total number of time slots *T* is small, it may not be possible to find the optimal deployment position in the end. When *T* is large, due to the adoption of the greedy strategy in DRL and the training error of the DNN, the UAV’s ability to maintain the optimal deployment position will be affected. This may cause the UAV to deviate from the optimal position and make exploratory movements to nearby positions. Suppose *T* has 200 time slots. After multiple rounds of training, the UAV identifies the optimal deployment position within 90–100 time slots. In the subsequent time slots, the UAV will either remain stationary at the optimal deployment position or exhibit slight fluctuations. However, when the reward obtained by the agent decreases, the UAV will try to return to the position with the maximum reward, resulting in the above-mentioned lingering phenomenon.

**Figure 7:**
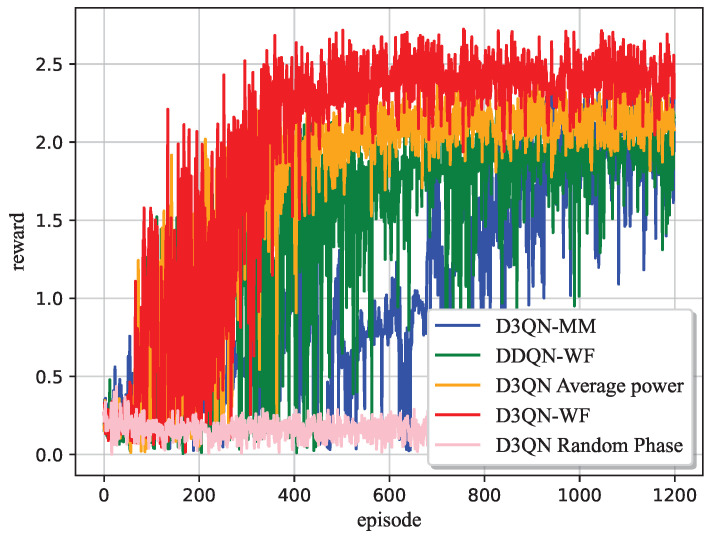
Reward of D3QN-WF.

Figure 7 shows the variation of rewards with the number of training rounds for four algorithms: DDQN-WF, D3QN with average power, D3QN-MM, and D3QN-WF. It can be seen from the figure that the reward value of the D3QN-WF algorithm tends to converge after approximately 500 training rounds, with only slight fluctuations occurring thereafter. This phenomenon arises due to minor deviations when the UAV reaches the optimal deployment position, as well as the continuous impact of dynamic environmental parameters on system performance. It is worth noting that compared to DDQN-WF, D3QN with average power and D3QN-MM, D3QN-WF exhibits a smaller convergence fluctuation range and more stable convergence characteristics. Comparative experiments with D3QN-WF without optimized phase angles further validate that optimizing the RIS phase parameters can significantly improve the system communication rate.

**Figure 8:**
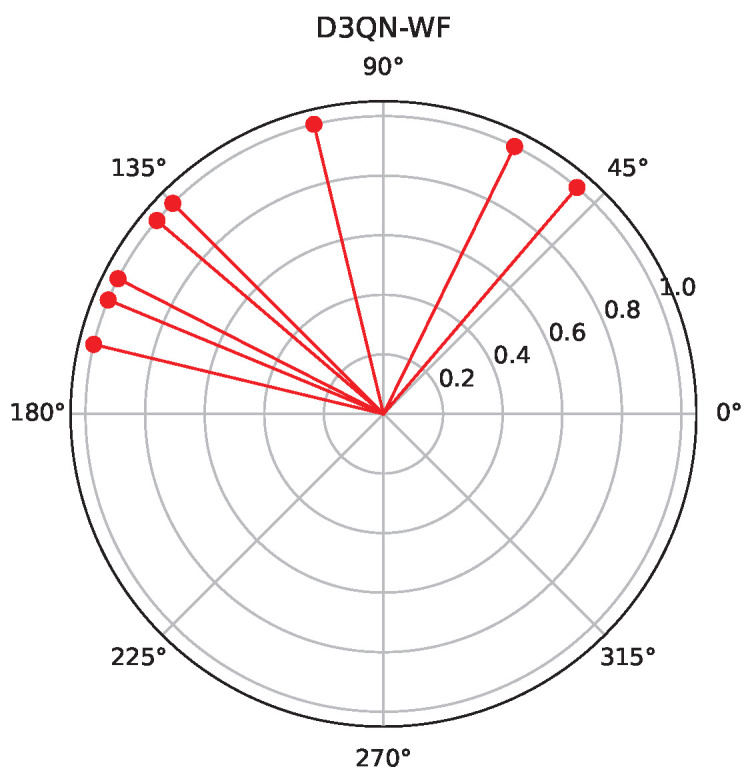
D3QN-WF phase.

**Figure 9:**
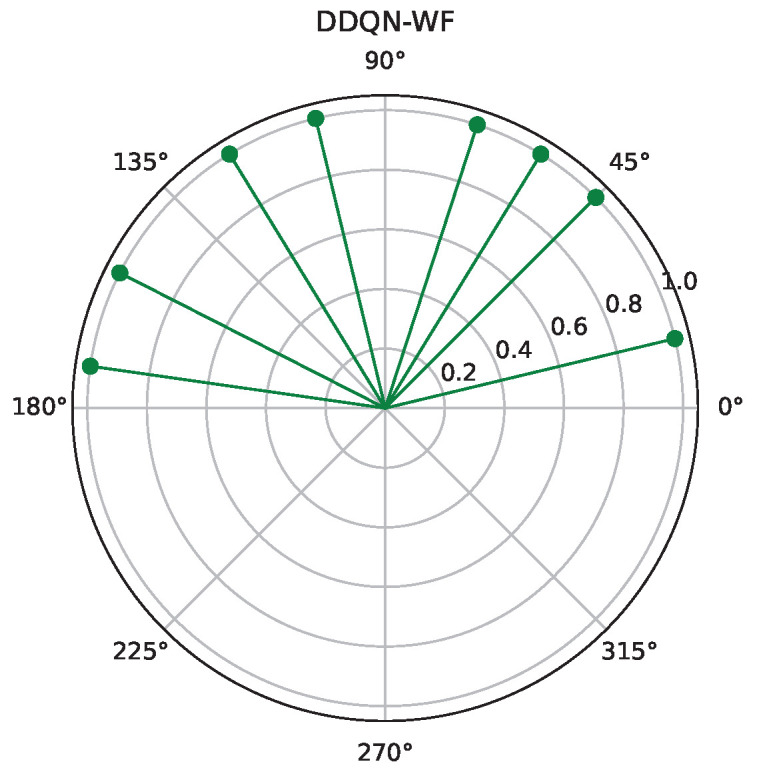
DDQN-WF phase.

Figure 8 and Figure 9, respectively, present the optimization results of two algorithms (DDQN-WF and D3QN-WF) for 8 RIS reflecting elements. Experiments show that the stability of D3QN-WF in dynamic environments is significantly superior to that of DDQN-WF. It can be seen from Figure 6 that multiple users are concentrated in the negative direction of the x-axis. Therefore, in comparison with these two algorithms, the phases optimized by D3QN-WF are more concentrated.

**Figure 10:**
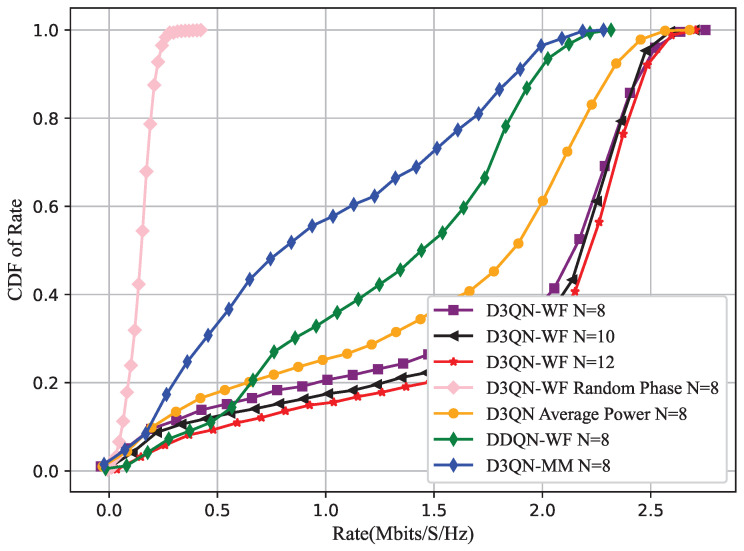
CDF of Rate.

Figure 10 presents the cumulative distribution function (CDF) of the system sum rate during the training process for several algorithms, including DDQN-WF, D3QN-WF with different numbers of RIS elements, D3QN-WF with unoptimized phase angles, D3QN-WF with average power, and D3QN-MM. Among them, a CDF value of 1 corresponds to the rate under the optimal 3D deployment coordinates of the UAV. The optimal deployment positions obtained by the algorithms are shown in Figure 6 as follows: the optimal deployment of the D3QN with average power algorithm is at (0 m, 130 m, 50 m); the optimal deployment of the DDQN-WF algorithm is at (70 m, 185 m, 50 m); and the optimal deployment of the D3QN-WF algorithm is at (0 m, 155 m, 50 m). In Figure 10, the CDF curves of DDQN-WF, D3QN with average power, and D3QN without optimized phase angles are located to the left of that of D3QN-WF, which illustrates the superiority of D3QN-WF and indicates that it has obvious advantages in the statistical distribution characteristics of the sum rate. The simulation results show that compared with DDQN-WF and D3QN-MM, the D3QN-WF algorithm increases the system sum rate at the optimal position by 15.9% and 17.6%, respectively. This is because the essence of D3QN lies in combining the decomposed structure of state value and advantage value in Dueling DQN with the dual-network structure of DDQN. This combination improves the accuracy of each action value estimation in D3QN and mitigates the overestimation issue inherent in traditional Q-learning. Therefore, D3QN-WF optimizes the RIS phase matrix and the UAV’s position more effectively. Meanwhile, it can be observed that reasonable power allocation also improves the overall system sum rate.

**Figure 11:**
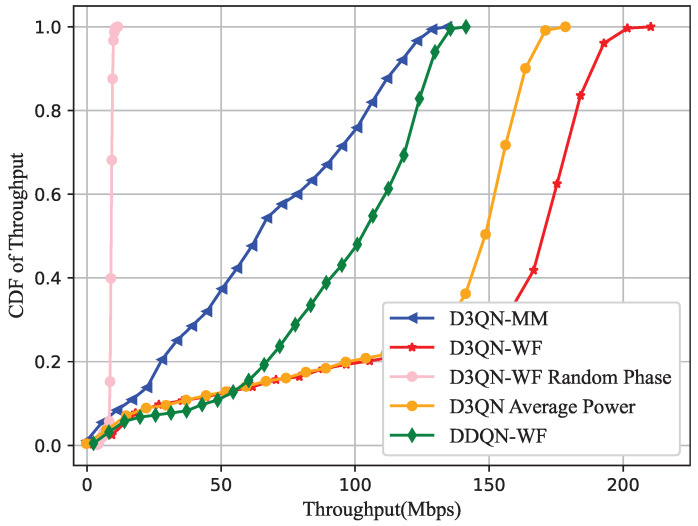
CDF of Throughput.

According to Equation (21), Figure 11 depicts the cumulative distribution function (CDF) of the system throughput $R_{sum}$ during the communication period. It can be seen that D3QN-WF improves throughput throughout the entire communication period compared to other algorithms. In Figure 6, for the DDQN-WF algorithm, although it can find a deployment position close to users during the optimization process, it overestimates the value of some actions. This overestimation leads to a longer time spent searching for the optimal position throughout the entire time slot *T*. As a result, the throughput during the entire time slot *T* is significantly lower compared to D3QN-WF. Therefore, it can be observed that throughout the entire communication period, due to the reasonable power allocation of the D3QN-WF algorithm, its throughput is 20% higher than that of D3QN with average power allocation, and total throughput is increased by 50.1% and 55.6% compared with DDQN-WF and D3QN-MM, respectively.

## 6. Conclusions

In this paper, research on the optimization of the RIS-assisted BS-UAV aerial-ground communication system is conducted. DRL is used to construct autonomous decision-making with deep interaction between the agent and the environment. The D3QN-WF and DDQN-WF algorithms were proposed to adjust the transmission power, reconstruct the RIS phase, and optimize the 3D coordinates to maximize the system sum rate. Simulation results show that, compared with the DDQN-WF algorithm, D3QN-WF has a faster convergence speed, which improves the system sum rate under the optimal deployment position. The total throughput during communication is increased by 50.1%, verifying the significant advantages of D3QN in dynamic environments and providing theoretical support for the optimization of future intelligent communication systems.

## Figures and Tables

**Table 1:** System Simulation Parameters.

Physical Meaning	Parameter	Value
Noise Power	$\sigma^{2}$	—80 dBm
Bandwidth	*B*	1 MHz
Path Loss Exponent	α	4
Path Channel Gain	β	—40 dBm
UAV Transmit Power	$P_{\mathrm{UAV}}$	30 dBm
Rice Factor	$\hat{R}$	$10^{3.3}$

**Table 2:** D3QN-WF Algorithm Hyperparameters.

Physical Meaning	Parameter	Value
Learning Rate	α	$10^{-3}$
Decay Factor	γ	0.9
Replay Buffer	*F*	1500
Greedy Policy	ε	0.1
Update Step	*O*	750
Mini-batch Size	Mini-batch size	75
Activation Function	Activation function	ReLU
Optimizer	Optimizer	RMSProp

## Data Availability

The data used to support the findings of this study are available from the corresponding author upon request.

## Abbreviations

The following abbreviations are used in this manuscript:

BS	base station
UAV	unmanned aerial vehicle
RIS	reconfigurable intelligent surfaces
ZF	zero-forcing
DRL	deep reinforcemen learning
6G	sixth Generation
5G	Fifth Generation
ML	Machine learning
RL	Reinforcement Learning
DDQN	Double Deep Q-Network
D3QN	Dueling Double Deep Q-Network
D3QN-WF	Dueling Double Deep Q-Network Water-Filling Algorithm
DDQN-WF	Double Deep Q-Network Water-Filling Algorithm
SINR	signal interference noise ratio
CSI	channel state information
LoS	line-of-sight
NLoS	non-line-of-sight
AoI	Area of Interest
MDP	Markov Decision Process
